# Therapeutic Plasma Exchange in Early-Onset Preeclampsia: A 7-Year Monocentric Experience

**DOI:** 10.3390/jcm12134289

**Published:** 2023-06-26

**Authors:** Antonella Iannaccone, Beatrix Reisch, Rainer Kimmig, Börge Schmidt, Laven Mavarani, Marvin Darkwah Oppong, Bartosz Tyczynski, Mark Dzietko, Michael Jahn, Alexandra Gellhaus, Angela Köninger

**Affiliations:** 1 Department of Gynecology and Obstetrics, University Hospital Essen, University of Duisburg-Essen, 45147 Essen, Germany; beatrix.reisch@uk-essen.de (B.R.); rainer.kimmig@uk-essen.de (R.K.); alexandra.gellhaus@uk-essen.de (A.G.); 2 Institute for Medical Informatics, Biometry and Epidemiology, University Hospital, University of Duisburg-Essen, 45147 Essen, Germany; boerge.schmidt@uk-essen.de (B.S.); laven.mavarani@uk-essen.de (L.M.); 3 Department of Neurosurgery and Spine Surgery, University Hospital Essen, University of Duisburg-Essen, 45147 Essen, Germany; marvin.darkwahoppong@uk-essen.de; 4 Department of Nephrology, University Hospital Essen, University of Duisburg-Essen, 45147 Essen, Germany; bartosz.tyczynski@uk-essen.de (B.T.); michael.jahn@uk-essen.de (M.J.); 5 Department of Pediatrics I, Division of Neonatology, University Hospital Essen, University of Duisburg-Essen, 45147 Essen, Germany; mark.dzietko@uk-essen.de; 6 Department of Gynecology and Obstetrics, St. Hedwig’s Clinic of the Order of St. John, University Regensburg, 93053 Regensburg, Germany; angela.koeninger@barmherzige-regensburg.de

**Keywords:** therapeutic plasma exchange, preeclampsia, soluble fms-like tyrosine kinase 1, placental growth factor, soluble endoglin

## Abstract

Different therapeutic apheresis techniques have been clinically tested to delay preterm delivery in the case of eoPE (early-onset preeclampsia). Our study evaluated the feasibility of TPE (therapeutic plasma exchange) compared to standard-of-care treatment. Twenty patients treated with 95 TPE sessions were included in the final analysis and retrospectively matched with 21 patients with comparable placental dysfunction. Gestational age at admission was 23.75 ± 2.26 versus 27.57 ± 2.68 weeks of gestation (WoG) in the control group (*p* = < 0.001), mean sFlt-1/PlGF ratio was 1946.26 ± 2301.63 versus 2146.70 ± 3273.63 (*p* = 0.821) and mean sEng was 87.63 ± 108.2 ng/mL versus 114.48 ± 88.78 ng/mL (*p* = 0.445). Pregnancy was prolonged for 8.25 ± 5.97 days when TPE was started, compared to 3.14 ± 4.57 days (*p* = 0.004). The median sFlt-1/PlGF Ratio was 1430 before and 1153 after TPE (−18.02%). Median sEng fell from 55.96 ng/mL to 47.62 mg/mL (−27.73%). The fetal survival rate was higher in TPE-treated cases. NICU (Neonatal Intensive Center Unit) stay was in the median of 63 days in the TPE group versus 48 days in the standard-of-care group (*p* = 0.248). To date, this monocentric retrospective study, reports the largest experience with extracorporeal treatments in eoPE worldwide. TPE could improve pregnancy duration and reduce sFlt-1 and sEng in maternal serum without impairing neonatal outcomes.

## 1. Introduction

Preeclampsia (PE) complicates 3 to 5% of pregnancies in Europe and is classified by new-onset hypertension defined as systolic blood pressure (SBP) ≥ 140 and/or diastolic BP (DBP) ≥ 90 mmHg) and proteinuria or evidence of end-organ damage (i.e., kidney, liver, central nervous system, or placenta) [1].

In case of SBP ≥ 160 and/or DBP ≥ 110 mmHg, HELLP (Hemolysis, Elevated Liver enzymes, and Low Platelets) syndrome [2] (defined according to the Tennessee Classification System as hemolysis with increased LDH > 600 U/L, AST ≥ 70 U/L, and platelets < 100 × 10^9^/L), pulmonary edema, renal insufficiency or new-onset cerebral or visual disturbances, severe PE occurred [3].

Classical early onset PE (eoPE) occurs before 34 WoG [4]. Clinically eoPE cases are often accompanied by fetal growth restriction (FGR) [3].

Hypotensive drugs and seizure prophylaxis with magnesium sulfate represent the actual cornerstones of the symptomatic PE treatment. Removing the placenta and, therefore, earlier termination of pregnancy is still the only causal therapy. Depending on the gestational week, this leads to preterm delivery and a high neonatal mortality and morbidity rate [5]. PE has short- and long-term consequences for maternal health. Short-term effects include hypertension and proteinuria. Long-term consequences on maternal health are the increased risk of hypertension, ischemic heart disease, and stroke [6,7].

While the underlying causes of PE are still controversial, clinical and pathological studies suggest that the placenta plays a crucial role in its development [8]. Due to insufficient trophoblast invasion in early pregnancy, placental dysfunction develops in later gestation [9]. The anti-angiogenic factors like soluble fms-like tyrosine kinase-1 (sFlt-1), the soluble receptor of the proangiogenic placental growth factor (PlGF), is one key factor in the development of symptoms in PE, causing endothelial dysfunction, and a predictive clinical biomarker in PE diagnosis [10]. Endoglin (Eng) (CD105) is a homodimeric transmembrane glycoprotein, a co-receptor for TGF (“transforming growth factor”)-β1 and -3, which is widely expressed on cell membranes of the vascular endothelium and syncytiotrophoblasts [11].

Eng has a pro-angiogenic activity that prevents apoptosis in hypoxic endothelial cells, and it is essential for endothelial nitric oxide (eNOS) activation [12].

Its soluble form, sEng, can be released from the placenta, has anti-angiogenic properties, and affects new vessels’ formation and vasodilation [10]. sEng, like sFlt-1, can cause endothelial dysfunction, which is responsible for most of the clinical manifestations of PE [10]. However, compared to sFlt-1, the data regarding its role in PE is sparse [13].

The discovery and characterization of these novel anti-angiogenic pathways involved in PE in the last decades have been particularly impactful in increasing understanding of the disease’s pathophysiology and directing predictive and therapeutic efforts [14].

Thus, therapeutical apheresis was approached to primarily eliminate the anti-angiogenic factors from maternal blood.

Our study aimed to analyze 24 cases of early severe PE treated with therapeutical plasma exchange (TPE) and elucidate its impact on maternal and neonatal outcomes compared to PE cases with an equivalent grade of placental dysfunction treated with standard-of-care.

## 2. Materials and Methods

This is a monocentric retrospective analysis of the use of TPE and the local standard-of-care in cases of eoPE with or without fetal growth restriction (FGR). In addition, patients treated with TPE were compared with a historical cohort of eoPE who did not receive TPE. TPE has been offered since 2014 at our tertiary perinatal center to preeclamptic patients <28 weeks of gestation at admission as an individual and experimental therapy approach after carefully counseling [15,16].

All participants or their legal guardians gave informed consent before inclusion into the study.

All methods were carried out following relevant guidelines and regulations and were in accordance with the Declaration of Helsinki.

The ethics committee approved the study at the University of Duisburg-Essen, University Hospital Essen, Germany (21-9898-BO).

Initially, the first 24 consecutive cases of eoPE treated with TPE were included in the retrospective analysis. The indications to offer TPE as an individual therapeutic option were the following:

- severe early-onset PE with clinical symptoms justifying immediate delivery
- high levels of sFlt-1/PlGF (>300) in very early gestational age (<28 WoG)
- no fetal doppler sonography findings that indicate delivery for fetal reasons (e.g., negative a-wave of the Ductus venosus)

**Figure 1:**
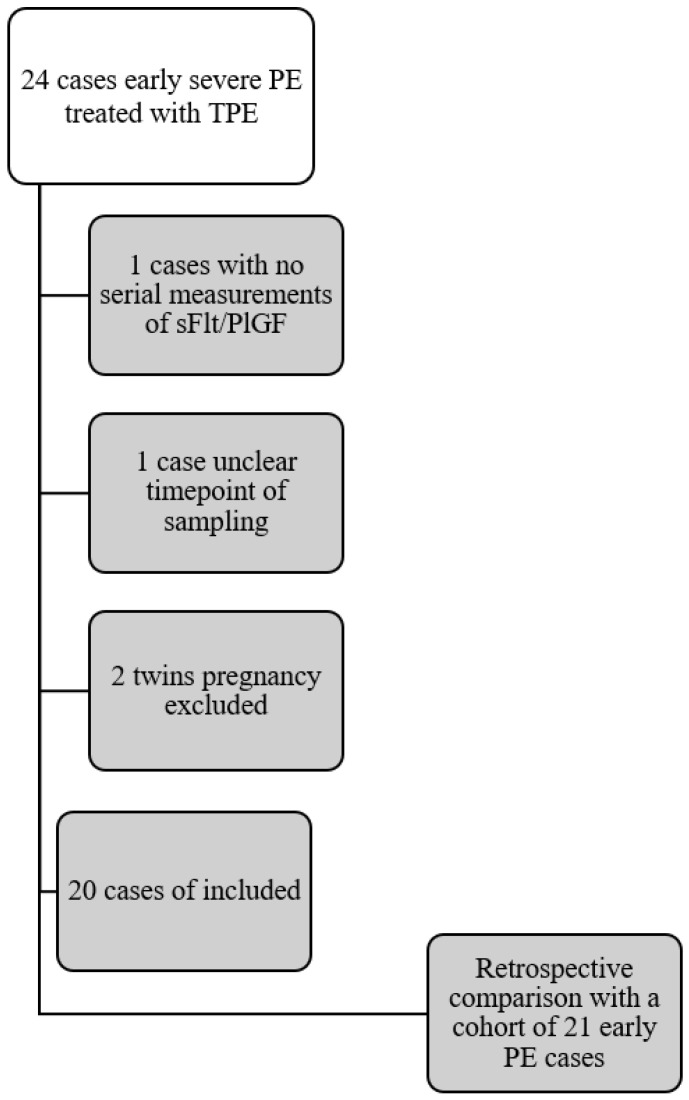
Flow Chart.

Four patients were excluded from the final analysis for the following reasons (s. Flow chart Figure 1):

- no serial measurements of angiogenic factors (1)
- unclear time points of blood sampling (1)
- twin pregnancies (2)

The standard-of-care control group included retrospectively selected patients with similar high sFlt-1/PLGF ratio levels with severe eoPE from our institution already described in other studies [17], which did not receive TPE.

Statistical analysis was performed using SPSS (version 27.0; SPSS Inc., Chicago, IL, USA) and GraphPad Prism (version 5.00; GraphPad Software Inc., San Diego, CA, USA).

For descriptive statistics, absolute and relative frequencies were calculated for categorical parameters, whereas continuous parameters were characterized using mean, standard deviation, median, minimum, and maximum.

The changes in the concentrations of angiogenic factors at admission, before and after the respective plasma separations, were recorded.

Wilcoxon tests were performed to compare continuous variables before and after TPE.

Clinical characteristics of the TPE-treated and of the untreated control group were compared using paired *t*-test. In addition, Mann-Whitney tests were performed to compare angiogenic factors and prolongation of pregnancy between the TPE treated and the control group.

The applied statistical tests were two-sided, and the results were considered statistically significant when *p* < 0.05.

Detailed methods are reported in Appendix A.

## 3. Results

### 3.1. Patient’s Characteristics

Our study cohort consists of 41 pregnant women with eoPE who were treated at the Department of Gynecology and Obstetrics, University Hospital Essen, Germany, between 2014 and 2021. 21 were treated according to the standard-of-care, constituting our non-treated control group, whereas 20 cases received additional TPE and served as our study group (Figure 1).

Demographic and clinical characteristics of the TPE-treated and untreated groups are reported in Table 1. Maternal age and the number of precedent pregnancies and deliveries did not differ between the groups. However, the mean BMI in patients treated by TPE was 33.87 ± 7.67 kg/m^2^, significantly higher than in patients not treated by TPE (18.80 kg/m^2^ ± 17.16, *p* = 0.003).

### 3.2. TPE Treatments and Maternal Characteristics

In total, 95 procedures were conducted, with a mean (±standard deviation, STD) of 4.60 ± 2.82 per patient, with a minimum of 1 and a maximum of 11.

TPE was started in the 24.25 ± 2.59 WoG, and patients stayed for 20.25 ± 9.83 days in the hospital (Table 1).

**Figure 2:**
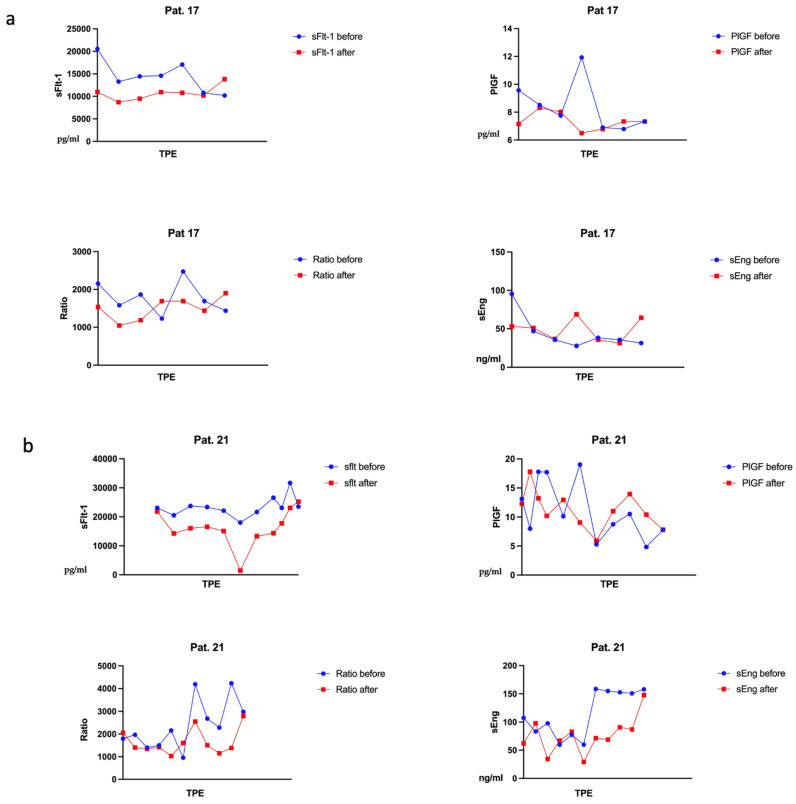

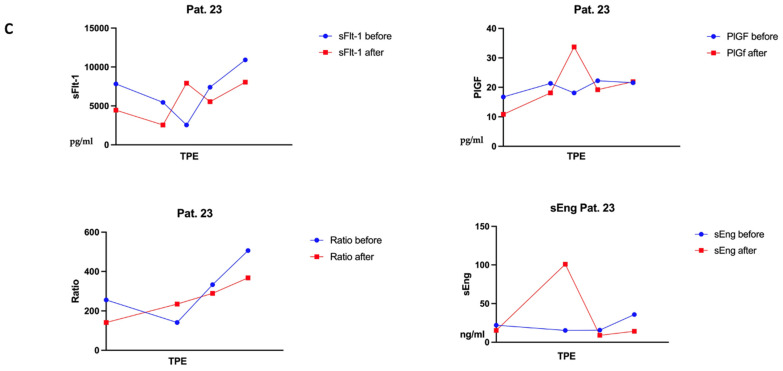
The course of angiogenic factors under TPE treatments of patient (**a**) 17, (**b**) 21, (**c**) 23.

Serial measurements of the angiogenic factors showed drops in the concentration of sFlt-1 and sEng after the procedure. The courses of the levels of cases 17, 21, and 23 are shown in Figure 2a–c. sFlt-1/PlGF remains stable during multiple TPE treatment courses. PlGF levels revealed a fluctuating course. Interestingly, we observed higher PlGF levels after TPE compared to before. Therefore, PlGF rises after TPE (Figure 2).

**Figure 3:**
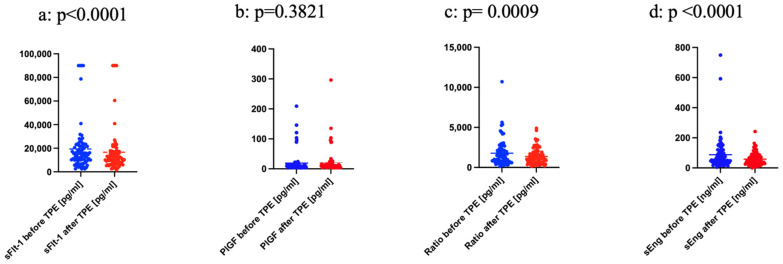
(**a**) sFlt-1 before and after TPE, (**b**) PlGF before and after TPE, (**c**) Ratio before and after TPE, (**d**) sEng before and after TPE.

As shown in Figure 3b, the difference in the levels of PlGF before and after TPE does not change significantly (*p* = 0.382). Conversely, after the TPEs, the levels of sFlt-1, sFlt-1/PlGF Ratio, and sEng are considerably lower, as shown in Figure 3a,c,d and Table 2.

The sFlt-1 concentration was 19406 pg/mL before and 16,624 pg/mL after the TPEs, and in the median, 14,865 pg/mL before and 11,015 pg/mL after treatments. sFlt-1 drops in the mean by −7.96% and in the median by −24.78% of total TPE treatments independent of single cases (Table 2). The difference is statistically significant (Figure 3a, *p* < 0.0001).

The difference between the sFlt-1/PlGF Ratio before and after the procedures remained significant (Figure 3c, *p* = 0.009): mean 1786 before and 1364 after TPEs, median 1430 before and 1153 after the TPEs (Table 2).

During the TPE procedures, 3 L of plasma were in median replaced: during the first 10 of the 95 procedures (10.52%), exclusively fresh frozen plasma (FFP) was used. Subsequently, a 4% human albumin solution was preferred (Table 3). 4% Human albumin was used in 34 of 95 procedures (35.79%). In 51 of 95 TPE (53.68%), we used two-thirds human albumin 4%, and one-third FFP compound. On average, 0.78 ± 1.12 L of FFP were replaced, and 2.38 ± 1.11 L of human albumin. The plasma flow was, on average, 41.49 ± 9.24 mL/min (Table 3).

**Figure 4:**
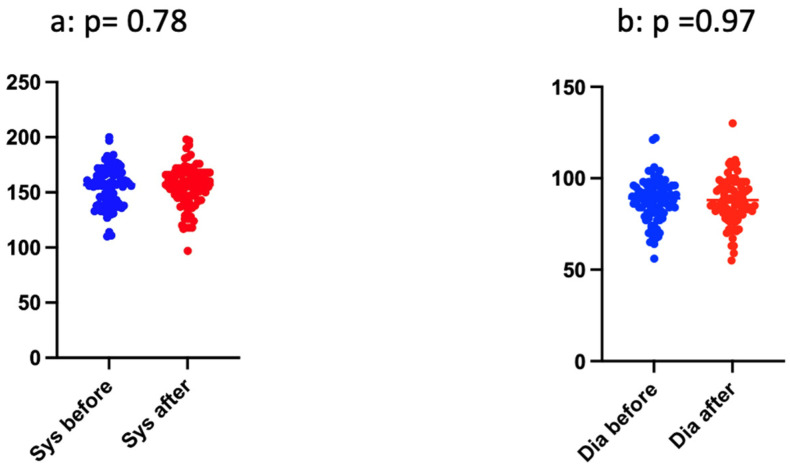
Blood pressure before and after TPE (**a**) systolic, (**b**) diastolic.

The blood pressure was stable during the procedures (Table 4 and Figure 4). The median systolic blood pressure value before and after TPE was 157 mmHg, and diastolic levels decreased from 89 to 88 mmHg.

**Figure 5:**
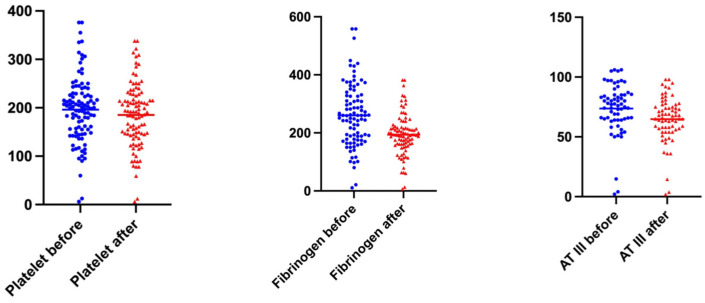
Laboratory before and after TPE: Wilcoxon Test Platelet, Fibrinogen and AT III *p* < 0.001.

The course of laboratory parameters (Table 5) showed slightly increased sodium and potassium levels. The levels of haptoglobin showed a median drop of −20.45%. Also, the levels of C reactive protein (−16.67%), LDH (−10.43%), total protein (−6.95%), platelets (−4.48%), and hemoglobin (−1.45%) dropped in the median. Leukocyte increase was observed by a median of 9.90% (Table 5). The coagulation tests also showed significant changes: AT III and fibrinogen dropped (in the median −25.38% and −12.26%), and INR (international normalized ratio) and pTT (activated partial thromboplastin time) increased (5.56% and 10.27%, Figure 5). A statistically significant difference in the levels before and after TPE for all the above parameters could be shown (Figure 5).

Regarding maternal complications, we had no severe adverse events but one case of hematoma after cesarean section, probably due to the depletion of the coagulation after TPE. One patient showed vaginal bleeding associated with low fibrinogen. Another patient had a placental abruption and very low levels of fibrinogen. However, this woman presented with an identical clinical problem in a further pregnancy without TPE. Two patients experienced transient hypotonia during two treatments, controlled with more fluid substitution. One patient experienced an initial allergic reaction after the start of FFP substitution. Hence, this situation was handled with antihistaminic treatment without sequelae.

### 3.3. Prolongation of Pregnancy, Fetal and Neonatal Outcome of TPE-Group Compared to Standard-Of-Care Control Group

**Figure 6:**
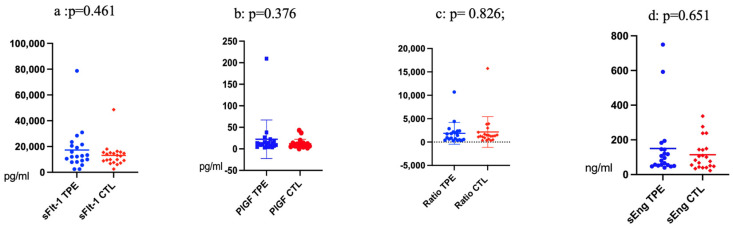
(**a**) sFlt-1 TPE versus non-TPE group; (**b**) PLGF TPE versus non-TPE group; (**c**): sFlt-1/PLGF Ratio TPE versus non-TPE group (*p* = 0.826); (**d**): sEng TPE versus non-TPE group (at admission).

The gestational week at PE onset was 23.75 ± 2.26 in cases treated by TPE. In contrast, in patients treated with standard-of-care, the WoG at admission was 27.14 ± 3.00 (*p* = 0.000). The gestational age in WoG at delivery was also significantly lower (25.45 ± 2.37) in cases treated with TPE (27.57 ± 2.77—*p* = 0.012—in the control group) (Table 1). The levels of angiogenic factors at presentation were not significantly different, with a mean sFlt-1/PlGF Ratio of 1946.26 ± 2301.63 in the treated group versus 2146.70 ± 3273.88 in the standard-of-care cases (*p* = 0.826, Figure 6c). Levels of sFlt-1, sEng, and PLGF also did not differ (Figure 6a,b,d).

We observed a significantly lower birthweight at delivery in cases treated with TPE of 622.37 ± 323.93 g and 892.14 ± 421.98 g in non-treated cases (*p* = 0.028). Percentiles were also different: in mean, 16.30 ± 11.53 in treated patients and 24.37 ± 16.54 in the eoPE fetuses in the control group (in the median 8th in the treated cases and 20th in the control group, *p* = 0.011, Table 1).

**Figure 7:**
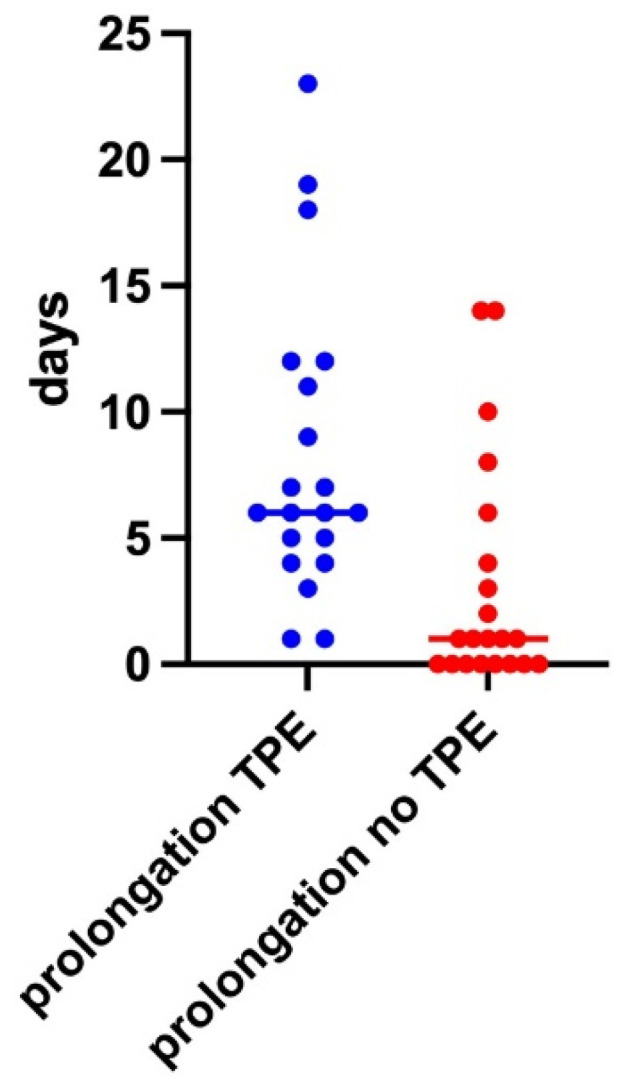
Prolongation of Pregnancy from admission for controls and from TPE Start in cases (*p* = 0.004).

Using TPE treatments significantly increased the prolongation of pregnancy (*p* = 0.004) compared to non-treated patients: on average, pregnancies complicated by eoPE and treated with TPE were prolonged for 8.25 ± 5.97 days, median 6 days, from the start of treatment. Instead, in the control group, delivery occurred at 3.14 ± 4.57 (median 1 day) after hospitalization (Figure 7).

In the case of eoPE, before 25 WoG, 11 women received TPE. In this group, 4 newborns survived, and 7 died (2 intrauterine death and 5 neonatal deaths).

3 pregnant women with eoPE before 25 WoG were treated after standard-of-care, and all newborns died (IUD, s. Table 6). When TPE was started after 25 WoG, all 9 newborns survived. 2 of the 18 newborns from eoPE-complicated pregnancies without the use of TPE died (IUD). In total perinatal mortality was 23.80% in the control group and 35% in the TPE group, with a survival rate after 25 WoG of 100% in the TPE group and 88.88% in the control group (Table 6).

Apgar values did not differ between the two groups: the mean Apgar at 1 min was 6.17 ± 2.01 in both groups, with a median of 7 and Apgar at 5 min was 7.56 ± 1.62 versus 8 ± 1.11(median 8 in both groups, *p* = 0.606), Apgar at 10 min was 8.39 ± 0.85 versus 8.53 ± 0.77, in median 9 in both groups (*p* = 0.520). In addition, umbilical cord blood gas analysis showed overall no statistically relevant difference between the two groups: arterial pH was in mean 7.30 ± 0.06 versus 7.28 ± 0.10, in median 7.30 versus 7.31 (*p* = 0.813); umbilical cord vein pH was 7.34 ± 0.05 versus 7.30 ± 0.11, in median 7.35 versus 7.32 (*p* = 0.351); Base Excess was 1.34 ± 4.38 versus −1.44 ± 3.71, in median 1.00 versus 0.00, *(p* = 0.100, Table 6).

The length of stay in the neonatal intensive care unit (NICU) was 70.22 ± 51.09 days in the newborns of women treated with TPE. In contrast, the NICU stay in the non-treated group without TPE was 63.83 ± 90.53 (in median 63 versus 48 days, *p* = 0.248).

The maximal respiratory support differs between the two groups: non-invasive ventilation was used in 3 preterm newborns of the TPE group and 13 of the non-TPE group. Conversely, invasive respiratory support was necessary for 14 TPE-groups neonates and 5 non-TPE neonates.

Regarding intraventricular hemorrhages, there were 5 cases in the TPE groups and 2 cases in the neonates from mothers of the control group; parenchymal bleedings were observed in 4 patients TPE-newborns, 2 of which were cerebellar, and 1 case of concomitant with a ventricular hemorrhage. In addition, one parenchymal bleeding was shown in 1 case of the non-TPE cohort.

## 4. Discussion

### 4.1. TPE in Pregnancy and PE

Early preeclampsia is a severe pregnancy complication affecting maternal and fetal health. This study investigates the experimental treatment of very early preeclampsia with the widespread available therapeutic plasma exchange (TPE) to reduce factors that may negatively affect preeclampsia. A low risk of morbidity and fatalities associated with plasmapheresis has been reported, but the incidence of these complications is not affected by pregnancy [18]. TPE is a frequently used and established extracorporeal clinical procedure during pregnancy for a large number of indications (e.g., TTP-thrombotic thrombocytopenic purpura, rhesus alloimmunizations, pancreatitis, aHUS-atypic hemolytic uremic syndrome, acute liver failure, catastrophic antiphospholipid syndrome, SLE-systemic lupus erythematosus and SLE-nephritis) [19].

Our data showed that TPE could significantly lower sFlt-1 and sEng levels and thus the sFlt-1/PlGF ratio. Interestingly, we observed higher PlGF levels after TPE than before. We hypothesize that removing sFlt-1 allows less binding of PlGF to sFlt-1, showing higher PlGF levels after sFlt-1 decreases. As a side effect, other laboratory parameters showed significant changes, i.e., C reactive protein levels, LDH, and coagulation tests, especially AT III and Fibrinogen. However, these changes did not result in clinical complications, except for one patient, who presented with vaginal bleeding at low fibrinogen levels. A further patient showed placental abruption associated with very low fibrinogen levels. Afterwards, we strictly replaced fibrinogen immediately to achieve levels of 200 mg/dL. Nevertheless, the patient with placental abruption and very low fibrinogen levels presented with the same clinical symptoms in a further pregnancy without TPE. We concluded that other reasons, but not TPE, were causing this worse condition twice.

Regarding pregnancy outcome, a prolongation of 8.25 days between the start of the treatment and birth could be achieved, with a maximum of 23 days.

This result contradicted the study by Martin et al. [20], who first described antepartum plasma exchanges for HELLP syndrome in seven women between 24 and 30 WoG with severe preeclampsia recruited for the treatment from 1984 to 1987. Maternal-fetal deterioration required cesarean delivery in all cases within 48 h after initiation of treatment^20^. The difference in our results could be related to the new technology and increased knowledge. Conversely, some studies demonstrated that therapeutic apheresis (TA) improved maternal outcomes in postpartum HELLP syndrome [21,22,23,24].

For the upcoming 30 years, no encouraging results were reported for applying plasma exchange therapy during pregnancy [20].

2011 Thadhani et al. [25] included eight women with eoPE in a pilot study who were the first to implicate this novel finding into clinical practice. Dextran sulfate cellulose apheresis treatments revealed reduced circulating sFlt-1 levels in maternal blood. In the following study 2016, they reported a reduction of 7–34% in sFlt-1 and a prolongation of gestation by 11 to 19 days in 11 PE cases [26].

Other authors used heparin-induced extracorporeal LDL precipitation (H.E.L.P.) in six early PE cases, postponing delivery by 15 days after the hospital admission. However, this method could not show a change in the sFlt-1 or PlGF serum levels. Positive effects on microcirculation and mitigation of endothelial damage through modulation of dyslipoproteinemia, pro-inflammatory, and rheological factors are supposed to mitigate PE, allowing pregnancy prolongation [27]. However, in 2018 a French Phase II trial with LDL-apheresis was interrupted after including two cases of severe PE without fetal growth restriction (FGR) because patients showed paradoxically increased sFlt-1 levels [28].

Our study’s mean prolongation of pregnancy is comparable with the studies mentioned above using DSA and LDL-apheresis but is in contrast to the French study. However, this result is quite surprising because different extracorporeal procedures are used. The gestational age in our study cohort is lower (i.e., ranging from 25 + 0 to 30 + 4 WoG in Thadani et al. 2016 and a mean ±SD of 25.6 ± 1 WoG in the study of Winkler et al. 2018). In addition, our patients showed a higher sFlt-1/PlGF Ratio than the above analysis (i.e., mean ±SD sFlt/PLGF = 664 ± 546 in the same study by Thadani 2016 and mean ±SD sFlt/PlGF = 551 ± 279 in Winkler et al., 2018). However, different measurement techniques (Roche^®^, Indianapolis, IN, USA versus Thermofisher ^®^, Waltham, MA, USA) cannot be excluded as a reason for the difference.

In a recent study by Gubensek et al., TPE and DSA are directly compared in a small cohort of six PE patients: the non-selective and widespread TPE was comparable to the DSA, allowing similar sFlt-1 reduction, and pregnancy was prolonged for 10 days after initiation of the procedures [29]. In addition, TPE was associated with fewer side effects (allergic reactions). These results were confirmed in the following analysis, including five PE cases before the 28th week of gestation treated exclusively with TPE [30]. The results of the studies of Gubensek and colleagues are in line with our observations.

However, contrary to the studies reported, our analyses concern a larger group of patients. In addition, we have not only measured the widely examined angiogenic factors sFlt-1 and PlGF and extended the study by including soluble endoglin levels. sEng showed similar results as sFlt-1, being partially eliminated from the circulation with the TPEs. Similar findings were reported by a case report in which, in a case of PE in the context of antiphospholipid syndrome, the use of TPE showed a reduction in sEng, and pregnancy was prolonged from 19 until 25 WoG [31].

Considering the studies above, more data is shown in favor of effectively removing sFlt-1 by using non-selective TPE than by using sFlt-1-specific columns. In a recent study by Matin et al. 2020, in ex vivo adsorption experiments using serum samples from patients with PE, scVEGF multimers reduced sFlt-1 levels by 85% and increased PlGF and VEGF levels by 20- and 9-fold, respectively [32]. However, these new columns still need to be tested in clinical applications. Furthermore, looking at the neonatal outcome in cases WoG < 25th, there was no survival in cases not treated by TPE, and all newborns whose mothers were treated by TPE after 25th WoG survived. The number of cases is low, so these results should be interpreted cautiously. Even if the mean WoG in TPE-treated patients was lower, no statistically significant difference in the NICU length of stay between the newborns of the treated and untreated patient groups could be registered. Similar observations are reported by Thadani et al., 2016. However, this study observed an improvement in lung function in neonates of treated women. In the authors’ opinion, this was not entirely surprising given that sFlt-1 has been directly implicated in the etiology of respiratory distress syndrome and bronchopulmonary dysplasia in preterm infants as shown in other studies [33,34].

However, invasive respiratory support was more often necessary in the TPE-treated group in our study. This could be explained by the lower gestational age at delivery in our TPE-treated patients. Also, more intracerebral bleedings were observed in the study group.

### 4.2. Meaning of the Study and Understanding Possible Mechanisms

Whether the targeted reduction of the anti-angiogenic factor sFlt-1 allows a prolonged pregnancy in preeclampsia or whether decreased sFlt-1 represents only an epiphenomenon of a more complex pathology associated with TPE cannot be answered by this study. Improvement of rheology and lipid metabolism, attenuation of inflammatory responses, and modulation of rejection-triggering immune responses may also be mediated by plasma exchanges, just like multiple conditions associated with abnormal factors such as toxins, antibodies or immune complexes can be addressed by TPE. In this respect, this study focuses primarily on evaluating the benefit-risk balance of plasma exchange in early preeclampsia. Plasmapheresis does not treat the underlying pathophysiology but may afford temporary improvement, allow restoration of homeostatic processes and prolong pregnancy to improve fetal outcome.

### 4.3. Strengths and Limitations

This is, so far, the most significant cohort of eoPE cases treated with TPE during pregnancy.

This is the first study to examine the elimination of sFlt-1/PlGF and sEng as anti-angiogenic factors, which are well-known as indicators of preeclampsia and placental dysfunction. Also, extensive analysis of laboratory parameters that changed before and after extracorporeal procedures are described. A further benefit of this study is comparing data with a historical control group of PE patients treated with standard-of-care treatment.

Moreover, a strength of this study is the examination of the neonatal outcomes in both the TPE-treated and control group since not all of the studies mentioned above included control groups (only Thadani 2016 and Winkler 2018). A limitation of the study is the retrospective character of the research and disparate study design with a different amount of TPE treatments per patient and a historically non-treated control PE group which is not matched per week of gestation and BMI. Since this is a non-randomised observational study, confounding or reverse causation cannot be excluded. Additionally, the indication of TPE and the timing of beginning the therapy were patient dependent. In some cases we started at the moment of worsening of the PE as an alternative to preterm birth. In other cases we started when we extensively high sFlt-1/PLGF ratios were measured at a very early gestational age. Therefore, the study demonstrates the feasibility and good tolerance of TPE in very early and severe PE beyond beneficial prolongation of pregnancy.

## 5. Conclusions

In this monocentric retrospective study cohort, we showed that therapeutic plasma exchange improved pregnancy duration in severe eoPE without adverse effects on pregnancy and neonatal outcomes. In addition, we demonstrated a significant reduction of the anti-angiogenic markers sFlt-1 and sEng in maternal serum after TPE. Moreover, other laboratory parameters showed significant changes without adverse clinical consequences for the patients.

Thus, TPE is a promising treatment option for lowering anti-angiogenic factors in maternal serum with the potential to prolong pregnancy and therefore improve neonatal outcomes. In extremely early PE, TPE was shown to prolong pregnancy towards higher pregnancy weeks, which allows neonatal survival, even when the prolongation was only around about one week.

Whether TPE or more selective apheresis procedures should be used for treating PE is still controversial.

TPE has applied safely also in pregnancy for other pathologies like thrombotic thrombocytopenic purpura or red blood cells alloimmunization. We provided with this study important validation for using this technique also for preeclampsia [19]. We cannot exclude that removing other unknown mediators next to the above described could also be the reason for pregnancy prolongation. TPE in early-onset severe preeclampsia can be offered as a personalized therapy, whereby close pregnancy monitoring during TPE treatment is necessary. Further research should better elucidate if the elimination of anti-angiogenic factors is decisive or if the modulation of other factors in addition or solely, such as rheological, lipidological, but also immunological factors, could contribute to the so far undiscussed modulation of symptoms, which allows a significant prolongation of pregnancy in studies using extracorporeal procedures in pregnancy complicated by eoPE. Also, further investigations should clarify if prognostic factors could determine which characteristics patients should present to benefit more from TPE than others who will not benefit from this therapy. The frequency and the optimal timing to start therapeutic plasma exchange still need to be clarified and should be elucidated in future studies.

## Figures and Tables

**Table 1:** Clinical characteristics at the admission of TPE-treated and non-TPE-treated pregnant women and outcome values. Data are presented as mean ± standard deviation. Comparisons have been statistically analysed with paired *t*-tests.

	TPE			Control			
	N	Mean	SD	N	Mean	SD	*p* Value
Age	20	31.95	6.92	21	32.52	5.55	0.771
WoG at admission	20	23.75	2.26	21	27.14	3	<0.001
Gravida	20	1.6	1.188	21	1.86	1.315	0.515
Para	20	0.7	1.13	21	0.48	0.873	0.48
BMI	15	33.87	7.67	20	18.8	17.16	0.003
WoG at Delivery	20	25.45	2.37	21	27.57	2.767	0.012
Birthweight	19	622.37	323.93	21	892.14	421.98	0.028
Percentile	10	16.3	11.53	14	24.37	16.54	0.173
sFlt-1at admission	20	17,218.5	16,521.89	21	13,153.55	9085.8	0.34
PlGF	20	20.35	44.81	21	11.77	10.37	0.413
sFlt-1/PlGF-Ratio	20	1946.26	2301.63	21	2146.7	3273.88	0.821
sEng	20	150	185.94	21	114.48	88.76	0.445
Time between TPE Start/Admission and delivery in days	20	8.25	5.97	21	3.14	4.57	0.004

**Table 2:** Angiogenic factors before and after TPE.

		Before TPE	Delta	Delta %	After TPE
sFlt-1 [pg/mL]	Median	14,865	−2537	−24.78	11,015
	Mean	19,406	−2491	−7.97	16,624
	SD	19,391	8659	43.84	18,946
PlGF [pg/mL]	Median	9.5	0.34	3.45	10.78
	Mean	19.1	−1.4	12.41	21.31
	SD	33.39	14.02	46.86	39.78
sFlt-1/PlGF	Median	1430	−142.8	−18.02	1153
	Mean	1786	−439.3	−9.08	1364
	SD	1613	1155	46.36	978.7
sEng [ng/mL]	Median	55.96	−10.62	−27.73	47.62
	Mean	87.63	−30.15	−2.72	58.24
	SD	108.2	92.77	101.8	43.05

**Table 3:** TPE-Treatments Parameters.

	Treatment-Volume [L]	Replaced with HA4% [L]	Replaced with GFPs [L]	Plasma Flow Min [mL/min]	Plasma Flow Max [mL/min]	LOHS	WoG Start TPE	N. of TPE Conducted in Each Patient
Median	3.00	3.00	0.00	42.00	46.00	17	23	3.50
Mean	3.19	2.39	0.78	41.49	46.56	20.25	24.25	4.6
Std. Deviation	0.4	1.11	1.12	9.25	7.08	9.83	2.59	2.82

**Table 4:** Blood pressure values during the TPE procedures.

		Before TPE [mmHg]	After TPE [mmHg]	Delta	Delta %	RR Highest during TPE [mmHg]
RR Sys	Minimum	110	97	−57	−13.40%	125
	Median	157	157	1.5	0.00%	167
	Maximum	200	198	49	1.01%	213
	Mean	155.1	155.2	0.03	−0.06%	164.6
	SD	17.86	18.93	18.26	−5.65%	16.9
RR dia	Minimum	56	55	−23	1.82%	48
	Median	89	88	0	1.14%	91
	Maximum	122	130	41	−6.15%	130
	Mean	87.68	88.05	0.37	−0.42%	90.21
	SD	11.28	12.41	12.21	−9.11%	10.62

**Table 5:** TPE Effect on laboratory parameters.

		Before TPE	After TPE	Delta	Delta %	*p*-Value
Thrombocytes [/nL]	No.	90	89	91		<0.0001
	Median	201	192	−17	−4.48%	
	Mean	202	190.8	−11.25	−5.54%	
	SD	59.83	59.3	28.57		
Hematocrit [%]	No.	91	89	92		<0.0001
	Median	0.39	0.349	−0.04	−11.20%	
	Mean	12.13	10.79	−1.3	−11.05%	
	SD	14.55	13.55	5.03		
Hemoglobin [g/dL]	No.	92	89	92		0.0092
	Median	10.35	10.2	−0.1	−1.45%	
	Mean	10.54	10.37	−0.21	−1.61%	
	SD	1.632	1.54	0.72		
LDH [U/L]	No.	85	85	83		0.0019
	Median	230	206	−11	−10.43%	
	Mean	238.7	215.6	−22.53	−9.68%	
	SD	66.91	53.56	63.46		
Haptglobin [g/L]	No.	79	80	74		<0.0001
	Median	0.66	0.525	−0.1	−20.45%	
	Mean	0.70	0.5306	−0.15	−24.51%	
	SD	0.42	0.3057	0.25		
CRP [mg/dL]	No.	33	26	23		0.0017
	Median	1.8	1.5	−0.3	−16.67%	
	Mean	1.95	1.708	−0.45	−12.32%	
	SD	1.20	0.9282	0.76		
Leucocytes [/nL]	No.	90	89	91		0.0165
	Median	11.01	12.1	0.5	9.90%	
	Mean	12.43	13.17	0.6	5.95%	
	SD	4.58	4.743	2.76		
Fibrinogen [mg/dL]	No.	80	79	74		<0.0001
	Median	260	194	−47	−25.38%	
	Mean	268.4	200.8	−62.61	−25.19%	
	SD	97.62	60.57	81.45		
AT III [%]	N0.	52	57	45		0.0029
	Median	77.5	68	−4	−12.26%	
	Mean	77.38	67.58	−6.89	−12.66%	
	SD	14.81	14.52	14.96		
INR	No.	86	88	87		<0.0001
	Median	0.9	0.95	0.04	5.56%	
	Mean	0.91	0.9534	0.04	4.57%	
	SD	0.07	0.07409	0.06		
pTT [s]	No.	87	88	88		<0.0001
	Median	26.3	29	1.45	10.27%	
	Mean	27.47	30.45	2.56	10.85%	
	SD	4.82	6.531	5.62		
Magnesium [mmol/L]	No.	8	5	5		0.8691
	Median	1.81	2.2	0	21.55%	
	Mean	2.04	2.082	0.09	1.86%	
	SD	1.27	1.376	0.18		
Natrium [mmol/L]	No.	92	87	89		<0.0001
	Median	137	139	2	1.46%	
	Mean	136.2	138.1	1.84	1.40%	
	SD	2.78	2.92	2.62		
Kalium [mmol/L]	No.	92	89	93		0.0036
	Median	4	4	0.1	0.00%	
	Mean	4.02	4.147	0.08	3.21%	
	SD	0.45	0.4617	0.56		
Protein [mg/dL]	No.	44	42	30		0.1096
	Median	5.18	4.82	−0.17	−6.95%	
	Mean	5.25	5.014	−0.13	−4.42%	
	SD	0.65	0.7998	0.39		

**Table 6:** Fetal outcome depending on pregnancy week and TPE versus standard of care.

	TPE Group (18)			Control Group (19)		
	Mean ± SD	Median	Min–Max	Mean ± SD	Median	Min–Max
Apgar 1	6.17 ± 2.00	7	2–9	6.17 ± 2.01	7	2–9
Apgar 5	7.56 ± 1.61	8	5–9	8 ± 1.11	8	5–9
Apgar 10	8.39 ± 0.85	9	6–9	8.53 ± 0.77	9	7–9
NapH	7.31 ± 0.06	7.3	7.2–7.4	7.28 ± 0.10	7.303	6.96–7.38
NvpH	7.34 ± 0.05	7345	7.25–7.45	7.30 ± 0.11	7.32	6.97–7.45
BE [mmol/L] TPE	1.34 ± 4.38	1	−5.5–13.2	−1.44 ± 3.71	0	−8.9–2.9
NICU-stay	70.22 ± 51.09	63	1–149	63.84 ± 90.53	48	1–412
(days)						
			No TPE		TPE	Total
WoG < 25 at delivery	survived		0		4	4
	not survived		3		7	10
	Total		3		11	14
WoG > 25 at delivery	survived		16		9	25
	not survived		2		0	2
	Total		18		9	27
All	survived		16		13	29
	not survived		5		7	12
	Total		21		20	41

## Data Availability

The data presented in this study are available on request from the corresponding author.

## Appendix A. Detailed Methods

### Appendix A.1. Patient’s Selection

The demographical and clinical data were obtained by anamnesis, physical examination, and medical record consultation. Data included maternal age, cigarette smoking during pregnancy, the gestational week at the time of blood sampling, maternal weight and height, and body mass index (BMI). In every case, an ultrasound scan was performed to calculate the estimated fetal weight and a doppler measurement to assess uteroplacental and fetal perfusion.

Pregnancy outcomes, including delivery mode, infant birth weight, gestational age at delivery, APGAR score and complications, including neonatal intensive care unit (NICU) admission, acidosis, placental abruption and maternal or fetal death, were recorded. In addition, neonatal outcomes like the length of NICU stay, intracerebral bleeding, respiratory support and mortality were recorded.

Preeclampsia was diagnosed following the ISSHP Guidelines [1]. by:

- de novo hypertension (>140 mmHg systolic or >90 mmHg diastolic) on at least two occasions measured 4 h apart in previously normotensive women after 20 weeks of gestation associated with signs of maternal organ dysfunction, including at least one of the following:
- proteinuria (>300 mg/day or a spot urine protein/creatinine ratio >30 mg/mmol),
- renal insufficiency (creatinine > 0.09 mmol/L or oliguria),
- liver disease (raised transaminases or severe right upper quadrant or epigastric pain),
- neurological problems,
- hematological disturbances (thrombocytopenia, disseminated intravascular coagulation, hemolysis),
- utero-placental dysfunction (fetal growth restriction, abnormal umbilical artery Doppler, or stillbirth).

FGR was diagnosed following the definition of the Delphi consensus [34]. Early FGR (<32 weeks) could be identified through either three solitary parameters: abdominal circumference (AC) <3rd centile, estimated fetal weight (EFW) <3rd centile and absent end-diastolic flow in the umbilical artery (UA), or four contributory parameters: AC or EFW < 10th centile combined with a pulsatility index (PI) > 95th centile in either the UA or uterine artery.

### Appendix A.2. Standard-of-Care Treatment of Preeclampsia

After admission, patients were monitored intensively and vital signs and blood pressure were repeatedly recorded. In addition, fluid intake and urine output were assessed. In addition, 24 h creatinine excretion was assessed if possible or replaced by albumin/creatinine ratio. In the case of oliguria, a urinary Foley catheter was used. Blood tests were repeated at least every 24 h to monitor kidney function, electrolytes, complete blood count, transaminases and bilirubin. In case of severe features of preeclampsia, patients received magnesium sulfate for eclampsia prophylaxis and fetal neuroprotection.

Antenatal corticosteroids for fetal lung maturation were applied depending on the week of gestation following local standards after counseling the patient. Oral antihypertensive treatment with oral methyldopa, nifedipine or metoprolol was used to control blood pressure. In case of persistent high blood pressure, intravenous urapidil or dihydralazine was used following German guidelines [35].

### Appendix A.3. Plasma Exchange Procedures

TPE was started if informed consent was given after a detailed explanation of the procedure. A double-lumen catheter was placed into a jugular vein.

TPE was conducted with Spectra Optia (Terumo BCT, Inc., Lakewood, CO, USA) centrifugal devices and COM.TEC (Fresenius, Bad Homburg, Germany). For anticoagulation of the extracorporeal circuit, a 3% acid citrate dextrose solution, USP Formula A (ACD-A) (Haemonetics, Munich, Germany), was added to the tubing sets during the apheresis procedures, with a regular anticoagulation ratio of 1:20. Plasma volume was estimated with the Kaplan formula (estimated plasma volume = (0.065 × weight [kg]) × (1-hematocrit)). The maximal plasma volume was limited to 4 L [36]. As there is no systematic data for plasma exchange in early-onset pre-eclampsia, our procedures are rather empirical. In the initial phase of the study, we replaced the extracted plasma volume with fresh frozen plasma (FFP). As treatment experience increased, the protocol was modified, and a 4% human albumin solution was primarily used. The Aim of this modification was a risk-reduction towards allergic reactions, especially transfusion-associated overload (TACO), transfusion-related lung injury (TRALI), transfusion-related immunomodulation (TRIM) and nosocomial infections, which are all critical issues related to the transfusion of plasma rich blood products [37]. At the University Hospital Essen, bleeding management during childbirth is guided by viscoelastic tests with corresponding algorithms for administering specific coagulation factor concentrates and hemostatic drugs. In this context, we estimated that prophylactic and preemptive plasma transfusions are more related to adverse events than patients’ benefits [37,38,39,40,41].

However, when fibrinogen fell below 150 mg/dL, we substituted only two-thirds of the total plasma volume with a 4% human albumin solution. To avoid spontaneous bleeding complications due to the depletion of clotting factors, the further third was substituted with FFP. To achieve a balance between the regeneration of endogenous proteins removed by TPE on the one hand, but also a sustainable reduction of pro-inflammatory and -coagulatory substances constantly produced by the placental pathology on the other hand, we performed the TPE every second day, if possible. Fresh frozen plasma (FFP) replaced the extracted plasma volume during the first treatments. As treatment experience increased, the protocol was modified, and a 4% human albumin solution was primarily used.

Throughout the procedure, complete monitoring of vital parameters, regular control of electrolytes and acid-base balance via blood gas analysis, and regular monitoring of plasmatic coagulation before and after the TPE was provided. The decision to deliver and discontinue TPE was based on maternal-fetal status judged by obstetricians. In addition, it considered the occurrence of HELLP syndrome, pathological fetal Doppler, abnormal CTG, pulmonary edema, or neurological symptoms.

### Appendix A.4. Collection and Analysis of Blood Samples

Blood samples were collected at admission, before and within 12 h of every TPE therapy or using S-Monovettes (Sarstedt AG & Co., Nümbrecht, Germany), stored at 4 °C, and processed within four h to avoid blood cell lysis. Blood fractionation is carried out by centrifugation for 10 min at 2500× *g* rounds per minute (rpm). Subsequently, three to four milliliters of the upper phase, constituting blood serum, are removed, stored at −80 °C, and subjected to the determination of sFlt-1, PlGF, and sEng.

### Appendix A.5. Determination of sFlt-1 and PlGF

50 μL of the pre-diluted sample plus 150 μL of dead volume was used to measure the concentration of sFlt-1 (BRAHMS sFlt-1 KRYPTOR assay, Cat. No. 845.075), PlGF-Plus (BRAHMS PlGF plus KRYPTOR assay, Cat. No. 859.075), all from Thermo Fisher Scientific, using BRAHMS KRYPTOR compact PLUS machine based on TRACE^®^ Technology (Time-Resolved Amplified Cryptate Emission) (Thermo Fischer Scientific, BRAHMS GmbH, Hennigsdorf, Germany), according to the protocol.

The detection limit was assessed as being 22 pg/mL for sFlt-1 and 3.6 pg/mL for PlGF. The functional assay sensitivity, detected by inter-assay precision of 20% coefficient of variability (CV), has been assessed as being lower than 29 pg/mL for sFlt-1 and 6.7 pg/mL for PlGF.

### Appendix A.6. Determination of sEng

To determine sEng levels, the Enzyme-linked Immunosorbent Assay (ELISA) kit for human Endoglin/CD105 (R&D Systems, Minneapolis, MN, USA, Cat. No. DNDG00)) was used following the manufacturer’s instructions. Samples were diluted based on previously measured sFlt-1/PLGF ratio (range: 1:5–1:100). Diluted serum samples were dispensed into the wells coated with anti-CD105 antibody and incubated for 2 h at room temperature, followed by incubation with a horseradish-peroxidase-conjugated antibody specific to CD105 for 2 h at room temperature. 200 microliter substrate solution containing 3,3′,5,5′-Tetramethylbenzidin was added for 30 min at room temperature, resulting in a yellow to blue color change. The degree of enzymatic turnover of the substrate was determined by dual-wavelength absorbance measurement at 450 and 620 nm as reference wavelength using an ELISA reader (TECAN, Model Sunrise; Austria GmBH, Grodig, Austria) and Magellan^TM^ 7 (TECAN, Männedorf, Switzerland) data analysis software. To determine sEng blood serum concentration levels, the non-linear regression model was used with a log/lin type of graph according to the instructions. The absorbance measured was directly proportional to the concentration level of sEng in the samples, which was calculated from the calibration curve. The results were expressed in ng/mL according to the established standard curve (0.156 ng/mL, 0.313 ng/mL, 0.625 ng/mL, 1.25 ng/mL, 2.5 ng/mL, 5 ng/mL, 10 ng/mL). The minimum detectable concentration of sEng was typically less than 0.030 ng/mL. Intra-assay variation was <4%, while interassay variation was <7%.
